# The consolidation paradox: rethinking thoracic radiotherapy in extensive-stage small cell lung cancer in the immunotherapy era

**DOI:** 10.3389/fonc.2026.2001564

**Published:** 2026-10-21

**Authors:** Patrizia Ciammella, Emanuele Alì, Federico Iori

**Affiliations:** Radiation Oncology Unit, Azienda USL-IRCCS di Reggio Emilia, Reggio Emilia, Italy

**Keywords:** artificial *Hippophae rhamnoides* forest, coal - mine reclamation area, ecological risk, partial least squares structural equation model, soil potentially toxic elements

## Abstract

The role of thoracic radiotherapy in extensive-stage small cell lung cancer (ES-SCLC) remains uncertain in the chemo-immunotherapy era. In the pre-immunotherapy era, the phase 3 CREST trial did not meet its primary overall-survival endpoint but showed reduced progression and higher 2-year overall survival rates with consolidative thoracic radiotherapy (cTRT). cTRT was not incorporated into the pivotal trials establishing chemo-immunotherapy as first-line treatment, leaving its contemporary role undefined. We reviewed the historical and contemporary evidence, grouping prospective studies by strategy: early concurrent radiotherapy during induction, post-induction consolidation during maintenance, low-dose immunomodulatory radiotherapy, and thoracic-only versus combined thoracic/extrathoracic irradiation. In the randomized phase 3 TRIPLEX trial, early concurrent radiotherapy during induction chemo-immunotherapy did not improve survival and was associated with more adverse events and non-progression deaths. In the randomized phase 2 TREASURE trial, conventional-dose cTRT during atezolizumab maintenance was associated with substantially higher rates of serious and fatal adverse events, together with marked lymphocyte depletion temporally associated with radiotherapy, although all patients had ECOG performance status 0–1; lower baseline DLCO was associated with fatal adverse events in an exploratory analysis. Single-arm studies (SAKK 15/19, a phase 2 adebrelimab study, and MATCH) did not report a comparable signal, but differences in design, dose, systemic agent, and adverse-event ascertainment preclude attributing this contrast to any single factor. A competing-risks analysis of TREASURE showed a numerically lower, non-significant cumulative incidence of overall radiologic progression with radiotherapy, with a numerically higher incidence of death without documented progression, and does not constitute evidence of reduced intrathoracic progression. Current evidence therefore does not support routine cTRT in ES-SCLC patients receiving chemo-immunotherapy, and conventional eligibility criteria may be insufficient to identify patients at risk of serious toxicity. We propose the term “Consolidation Paradox” for the hypothesis that improved systemic control may increase the value of residual thoracic disease control while narrowing the safety margin for thoracic irradiation. The ongoing randomized RAPTOR/NRG-LU007 trial will address the efficacy of post-induction consolidation; future trials should also record immune-cell dosimetry, stratify by pulmonary reserve, report thoracic progression separately, and analyze progression and death without progression as competing risks.

## 1 Introduction

Small cell lung cancer (SCLC) accounts for approximately 15% of all lung cancers and is among the most radiosensitive solid malignancies (1). In limited-stage disease, thoracic radiotherapy combined with platinum-based chemotherapy remains a cornerstone of curative-intent treatment (2). In extensive-stage disease (ES-SCLC), systemic therapy is the mainstay of treatment: IMpower133 and CASPIAN established atezolizumab and durvalumab, respectively, in combination with platinum-etoposide as first-line treatment (4, 5). Neither trial incorporated thoracic radiotherapy (TRT) as a protocol-defined component, leaving its role in the contemporary setting untested. The systemic landscape has continued to evolve: the addition of lurbinectedin to atezolizumab maintenance improved outcomes in the phase 3 IMforte trial (9), and the DLL3-directed bispecific T-cell engager tarlatamab has been approved after platinum-based therapy (10).

Two observations keep the question of thoracic radiotherapy open. Intrathoracic progression remains the dominant pattern of failure after first-line chemo-immunotherapy (6), and retrospective series have associated TRT with improved outcomes in this setting (7). These series, and a meta-analysis that pooled them (8), are exclusively non-randomized; they are subject to selection and immortal-time biases and cannot establish a causal survival benefit. Prospective studies have since combined TRT with chemo-immunotherapy, but they evaluated distinct strategies, differing in timing relative to induction, dose, target extent, and population, and reported divergent safety findings (11–14, 23, 33). An invited editorial accompanying the publication of TREASURE framed the tension between thoracic irradiation and checkpoint blockade as a question of sequencing (18), and published correspondence has added methodological detail (15–17).

In this narrative review, we summarize the historical and contemporary evidence, grouping prospective studies by treatment strategy rather than treating them as variants of a single “consolidative” intervention. We then consider patient selection, timing, dose and fractionation, and treatment volume as candidate determinants of the therapeutic index, not as established predictors, and discuss the “Consolidation Paradox”: the hypothesis that improved systemic control may increase the relevance of residual thoracic disease while making the safe integration of thoracic irradiation more difficult. This framework is intended to organize the available evidence and generate testable hypotheses rather than to provide a clinical recommendation.

## 2 Materials and methods

### 2.1 Approach to the literature

This narrative review was designed to synthesize the historical and contemporary evidence on thoracic radiotherapy in extensive-stage small cell lung cancer (ES-SCLC), with particular emphasis on its integration with modern systemic therapy and immune checkpoint blockade. We searched PubMed/MEDLINE and Embase for publications from January 2015 through September 16, 2026. The search included terms related to ES-SCLC, thoracic radiotherapy, consolidation, immunotherapy, immune checkpoint inhibitors, and treatment-related lymphopenia, together with the names of relevant systemic therapies and prospective trials. Reference lists of key publications were screened for additional studies, including earlier landmark randomized trials, and targeted searches using individual trial names were performed when appropriate. Conference abstracts and presentations from major international oncology meetings, including ASCO, ESMO, and the World Conference on Lung Cancer, were also reviewed, with particular attention to studies reported between 2024 and 2026. ClinicalTrials.gov was searched for prospective trials evaluating thoracic radiotherapy in combination with first-line or maintenance systemic therapy. Published correspondence and letters to the editor were considered when they provided additional methodological or clinical interpretation of relevant prospective studies. Randomized and prospective trials, retrospective cohorts, and relevant systematic reviews or meta-analysis were considered according to their contribution to the questions addressed in this review, including efficacy, safety, patient selection, treatment timing, dose and fractionation, and treatment volume. As this was a narrative review, no formal systematic-review protocol, meta-analytic framework, risk-of-bias assessment, or grading of the strength of evidence was applied; candidate selection factors (**Table 1**) are therefore listed descriptively according to the type of supporting evidence. Studies were selected and interpreted according to their relevance to the clinical and biological questions underlying the proposed therapeutic-index framework.

**Table 1:** Candidate factors for selecting patients for thoracic radiotherapy, listed by type of supporting evidence.

Candidate factor	Principal source(s)	Type of evidence	Proposed role in future trials
Response to induction therapy	CREST, Jeremic et al., TREASURE, SAKK 15/19 (3, 12, 13, 30)	Eligibility criterion in prospective trials	Eligibility/stratification
Performance status	CREST, TREASURE, SAKK 15/19 (3, 12, 13)	Eligibility criterion (ECOG 0–1 in both post-induction studies); does not explain cross-trial safety differences and does not capture functional reserve	Eligibility/stratification
Residual thoracic disease	CREST (3)	*Post hoc* subgroup analysis	Stratification
Number of distant metastases/extrathoracic control	CREST secondary analysis, RTOG 0937, retrospective series (26, 31, 32)	*Post hoc* analysis; randomized phase 2; retrospective	Stratification
Liver and brain metastases	Wróbel et al. (27)	Meta-analysis of non-randomized studies	Exploratory
Pulmonary reserve (DLCO)	TREASURE (12)	Relatively reduced baseline DLCO in the study population; lower DLCO among fatal cases in the TRT arm (exploratory, six events)	Stratification; distinct from performance status
Target volume/completeness of consolidation	TREASURE, Ninia et al. (12, 26)	Exploratory; retrospective	Exploratory
Dose to circulating immune cells (EDRIC)	Feng et al., TREASURE (12, 25)	Retrospective; exploratory	Prospective dosimetric variable
Molecular subtype, ctDNA, and immune biomarkers	Adebrelimab study (33)	Exploratory, single-arm; otherwise untested in this setting	Translational endpoint

Factors are listed descriptively according to the type of supporting evidence; no formal grading of evidence strength or direction of association was performed.

## 3 Historical rationale for thoracic consolidation

### 3.1 Pre-immunotherapy randomized evidence

Three randomized studies define the pre-immunotherapy evidence base, and they differ in design in ways that matter for interpretation. Jeremic et al. enrolled patients who, after three cycles of cisplatin-etoposide, had a complete response at distant sites and at least a partial response in the thorax, and randomized them to accelerated hyperfractionated thoracic radiotherapy (54 Gy in 36 fractions over 18 treatment days) with concurrent carboplatin-etoposide followed by further chemotherapy, or to further cisplatin-etoposide alone (30). Median overall survival was 17 versus 11 months in favor of radiotherapy. This single-institution trial selected a highly favorable population with complete extrathoracic response and used a substantially higher biologically effective dose than later trials; its results therefore cannot be directly extrapolated to less selected populations.

CREST randomized 498 patients who had responded to first-line platinum-based chemotherapy to prophylactic cranial irradiation with or without thoracic radiotherapy (30 Gy in 10 fractions) (3). The primary overall-survival endpoint was not met: 1-year overall survival was 33% with cTRT versus 28% without cTRT (HR, 0.84; 95% CI, 0.69–1.01; P = .066). However, cTRT reduced the risk of progression (HR, 0.73; 95% CI, 0.61–0.87; P = .001), and 2-year overall survival was 13% versus 3% (P = .004). Subgroup analysis suggested less benefit in patients with a complete response or without residual intrathoracic disease (3). A subsequent secondary analysis, based on additional data on number and sites of metastases collected for 260 patients from the nine highest-accruing centers, suggested that future trials of more intensive thoracic and extrathoracic radiotherapy should focus on patients with fewer than three distant metastases (31). These analyses were *post hoc* and restricted to a subset of the trial population, and should be regarded as hypothesis-generating rather than as validated selection criteria.

NRG Oncology RTOG 0937 addressed a different question: whether extending consolidation beyond the thorax improves survival (32). Patients with one to four extracranial metastases responding to chemotherapy were randomized to prophylactic cranial irradiation alone or with consolidative radiotherapy (45 Gy in 15 fractions) to the thorax and residual metastases. The trial crossed its futility boundary at interim analysis and closed after 97 patients had been randomized; 1-year overall survival was 60.1% with cranial irradiation alone and 50.8% with consolidative radiotherapy (P = .21), although consolidative radiotherapy delayed progression. RTOG 0937 therefore shows that improved disease control with more extensive consolidation does not necessarily translate into improved survival. A meta-analysis of the pre-immunotherapy evidence suggested a possible survival benefit of thoracic radiotherapy, although heterogeneity in patient selection and treatment limits this conclusion (22).

## 4 Thoracic radiotherapy in the immunotherapy era: distinct strategies

The prospective studies combining thoracic radiotherapy with chemo-immunotherapy should not be regarded as variants of a single “consolidative” intervention. They tested strategies that differ in intent, in timing relative to induction and response assessment, in dose, and in target extent. We therefore group them into four categories (**Table 2**, **Figure 1**). Comparisons across categories are presented only as context and should not be interpreted as comparative evidence. In this review, the term cTRT is reserved for post-induction strategies.

**Table 2:** Prospective studies combining thoracic radiotherapy with chemo-immunotherapy in ES-SCLC, grouped by treatment strategy.

Strategy	Study (design, n)	Systemic therapy	TRT timing	Dose	Population	Key safety finding	Efficacy
Early concurrent	TRIPLEX (randomized phase 3, n=228) (11)	Platinum-etoposide + durvalumab	Started 21–28 days after C1D1	30 Gy/10 fx	Randomized at start of first-line therapy, before response assessment	More AEs; more non-progression deaths (14.8% vs 3.5%); early discontinuation	No OS or PFS benefit (OS HR 1.12; PFS HR 1.09)
Early concurrent	TROG 20.01/CHEST-RT (single-arm phase 2) (23)	Platinum-etoposide + durvalumab	With cycle 3 or 4 (sequential if not feasible)	30 Gy/10 fx	Thoracic disease encompassable in a RT field	Primary endpoint: safety/feasibility	Results awaited
Post-induction	TREASURE (randomized phase 2, n=68) (12)	Atezolizumab maintenance	During maintenance	30 Gy/10 fx	ECOG 0–1; ≥SD after induction; relatively reduced baseline DLCO	SAE 61.3% vs 18.2%; fatal AEs 19.4% vs 3.0%; persistent lymphopenia	OS HR 1.55 (ns); numerically lower, non-significant CIF of overall progression (80.6% vs 90.6%)
Post-induction	SAKK 15/19 (single-arm phase 2, n=46) (13)	Durvalumab maintenance	Within 5 weeks of induction	39 Gy/13 fx	ECOG 0–1	23.9% G3–4 TRAE (mainly hematologic); no severe TRT-specific toxicity	12-mo PFR 15.6% (primary endpoint not met)
Post-induction	Adebrelimab study (single-arm, single-center phase 2, n=67; 45 irradiated) (33)	Adebrelimab + platinum-etoposide, then maintenance	Sequential after 4–6 cycles, responders only	≥30 Gy/10 fx or ≥50 Gy/25 fx	ECOG 0–1; responders	No treatment-related deaths; G≥3 pneumonitis 7% in irradiated patients	Median OS 21.4 mo (all patients); non-randomized
Post-induction, thoracic ± extrathoracic	RAPTOR/NRG-LU007 (randomized phase 2/3, ongoing) (34, 35)	Atezolizumab maintenance	First 5 weeks of maintenance	20, 30 or 45 Gy to up to 5 sites	SD or PR after 4–6 induction cycles; ECOG 0–2	Toxicity comparison (secondary)	PFS (phase 2) and OS (phase 3); temporarily closed to accrual; results awaited
Low-dose immunomodulatory	MATCH (single-arm phase 2, n=56) (14)	Platinum-etoposide + atezolizumab	Days 1–5 of cycle 1	15 Gy/5 fx	Selected; ECOG 0–1	Mainly hematologic toxicity	ORR 87.5%; median PFS 6.9, OS 16.9 mo

TRIPLEX data are from a conference presentation. AE, adverse event; C1D1, cycle 1 day 1; fx, fractions; ns, not significant; PFR, progression-free rate; SD, stable disease; PR, partial response; TRAE, treatment-related adverse event.

**Figure 1:**
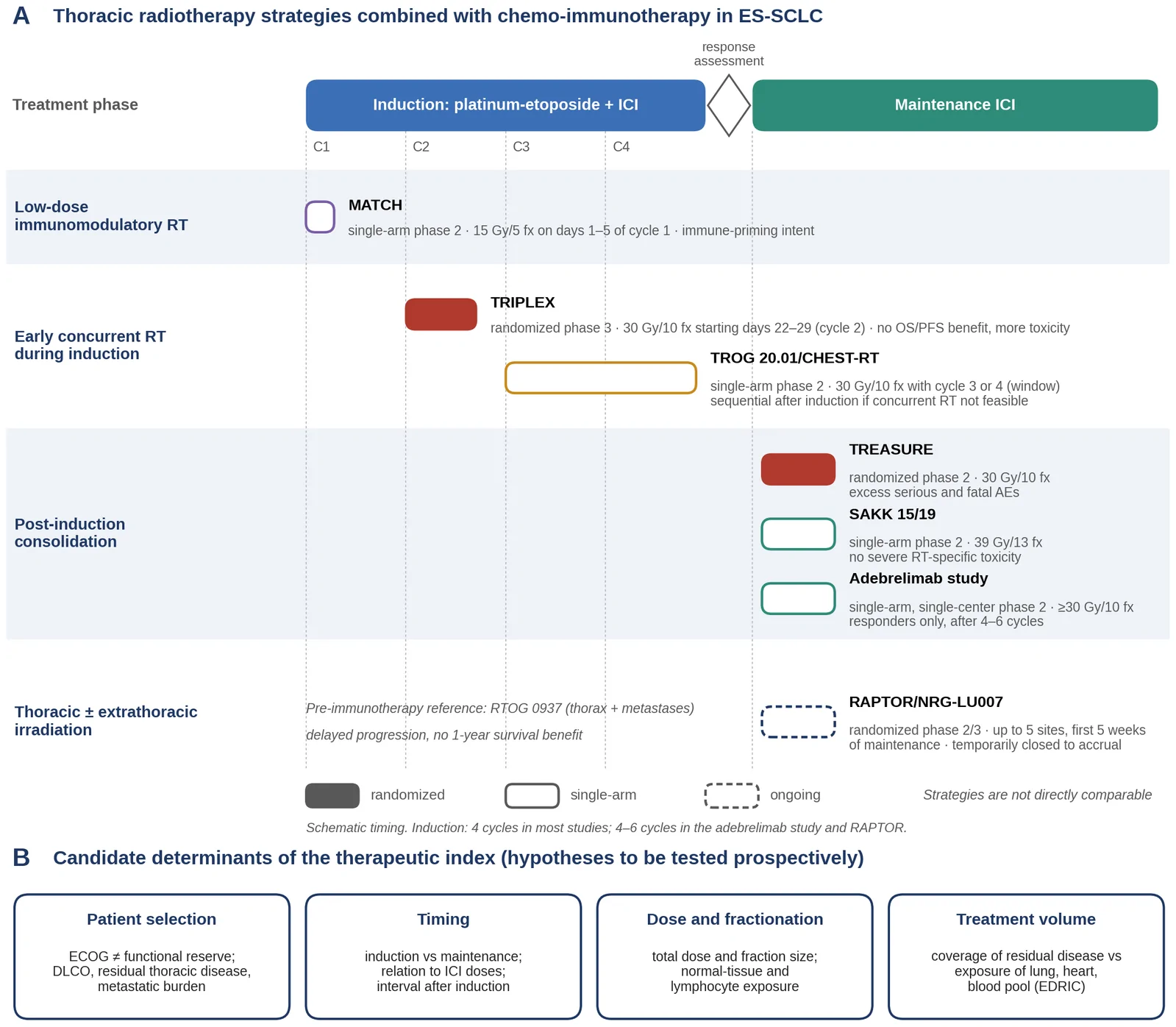
Thoracic radiotherapy strategies combined with chemo-immunotherapy in ES-SCLC and candidate determinants of the therapeutic index. **(A)** Prospective studies positioned according to the timing of thoracic radiotherapy relative to induction chemo-immunotherapy and maintenance immune checkpoint inhibition; filled bars indicate randomized trials, open bars single-arm studies, and dashed bars ongoing trials. Bar positions are schematic and reflect protocol-specified timing: radiotherapy on days 1–5 of cycle 1 (MATCH), starting 21–28 days after day 1 of cycle 1 (TRIPLEX), concurrent with cycle 3 or 4, with a sequential option (CHEST-RT), and after completion of induction during maintenance (TREASURE, SAKK 15/19, adebrelimab study, RAPTOR). RTOG 0937 is shown as a pre-immunotherapy reference for combined thoracic and extrathoracic irradiation. The strategies differ in intent, timing, dose, target extent, and population and should not be compared directly. **(B)** Candidate determinants of the therapeutic index proposed in this review, regarded as hypotheses to be tested prospectively rather than as established predictors. AE, adverse event; C, cycle; DLCO, diffusing capacity for carbon monoxide; ECOG, Eastern Cooperative Oncology Group performance status; EDRIC, estimated dose delivered to circulating immune cells; fx, fractions; ICI, immune checkpoint inhibitor; OS, overall survival; PFS, progression-free survival; RT, radiotherapy.

### 4.1 Early concurrent thoracic radiotherapy during induction

TRIPLEX was a randomized phase 3 trial in which 228 patients from 20 European centers were randomized at the start of first-line treatment to platinum-etoposide plus durvalumab with or without concurrent thoracic radiotherapy (30 Gy in 10 fractions), starting 21–28 days after day 1 of the first cycle (11). Because randomization preceded response assessment, the population included patients who would subsequently not respond or progress, unlike studies that randomize or treat after induction. The trial was discontinued because of excess serious adverse events. Radiotherapy did not improve overall survival (median 10.0 vs 11.1 months; HR, 1.12; 95% CI, 0.82–1.54) or progression-free survival (HR, 1.09). Deaths from causes other than disease progression were more frequent with radiotherapy (14.8% vs 3.5%), a difference that was no longer statistically significant when deaths occurring before the start of radiotherapy were excluded (9.6% vs 3.5%; P = .067). As the only completed randomized phase 3 trial of this strategy, TRIPLEX provides the strongest available evidence that early concurrent conventional-dose thoracic radiotherapy during induction chemo-immunotherapy does not improve survival and may increase toxicity; it does not address post-induction consolidation. TROG 20.01/CHEST-RT evaluates 30 Gy in 10 fractions concurrent with cycle 3 or 4, with a sequential option when concurrent treatment is not dosimetrically feasible (23); it is single-arm, and mature results are awaited.

### 4.2 Post-induction thoracic consolidation during maintenance

TREASURE randomized 68 patients with ECOG performance status 0–1 and at least stable disease after induction to atezolizumab maintenance with or without thoracic radiotherapy (30 Gy in 10 fractions); it was stopped early for excess fatal adverse events, and its findings are discussed in Section 5 (12). SAKK 15/19 delivered 39 Gy in 13 fractions during durvalumab maintenance in 46 patients with ECOG performance status 0–1 (13); no severe toxicity attributable to thoracic radiotherapy was reported, but the primary endpoint was not met (12-month progression-free rate, 15.6% versus a target of 25%). In a single-center, single-arm phase 2 study, 67 patients received adebrelimab plus platinum-etoposide, and responders were offered sequential involved-field thoracic radiotherapy (≥30 Gy in 10 fractions or ≥50 Gy in 25 fractions) with adebrelimab maintenance; 45 patients were irradiated (33). Median overall survival was 21.4 months, and no treatment-related deaths occurred. This study cannot establish the efficacy of radiotherapy: radiotherapy was not randomized and was given only to responders, so the difference reported between irradiated and non-irradiated patients is confounded by response and selection. Within this category, the divergent safety profiles of TREASURE and the single-arm studies cannot be attributed to a single factor, because the studies differed in design (randomized versus single-arm), dose, systemic agent, target volume, and adverse-event ascertainment.

### 4.3 Low-dose immunomodulatory thoracic radiotherapy

MATCH tested a biologically distinct concept: low-dose radiotherapy (15 Gy in 5 daily fractions) delivered on days 1–5 of the first cycle of induction platinum-etoposide plus atezolizumab, followed by atezolizumab maintenance, with the intent of immune priming rather than cytoreductive consolidation of residual disease (14). Its 3-year analysis in 56 patients reported an objective response rate of 87.5%, a median PFS of 6.9 months, and a median OS of 16.9 months, without a safety signal comparable to that of TRIPLEX or TREASURE. Because MATCH was single-arm, enrolled a selected population, and pursued a different therapeutic intent, it should not be used to infer the safety or efficacy of consolidative-dose strategies.

### 4.4 Thoracic-only versus combined thoracic and extrathoracic irradiation

CREST, TREASURE, and SAKK 15/19 treated the thorax only, whereas RTOG 0937 irradiated thoracic and residual extrathoracic disease and found delayed progression without improved survival (32); Jeremic et al. restricted thoracic radiotherapy to patients whose extrathoracic disease had completely responded (30). These approaches differ in treated volume, cumulative normal-tissue and immune-cell exposure, and the population selected. In the chemo-immunotherapy era, evidence on comprehensive consolidation of oligo-residual disease is limited to retrospective series (26). RAPTOR/NRG-LU007, which allows irradiation of up to five thoracic and extrathoracic sites during atezolizumab maintenance, is the key ongoing randomized trial evaluating post-induction consolidative radiotherapy in this setting (34).

## 5 What TREASURE adds: safety, biology, and competing risks

TREASURE provides the most detailed randomized assessment of thoracic radiotherapy during checkpoint-inhibitor maintenance (12). Serious adverse events occurred in 61.3% of patients receiving radiotherapy versus 18.2% without radiotherapy, and fatal adverse events in 19.4% versus 3.0%. Causal attribution of individual deaths was not straightforward: treating investigators considered some fatal events unrelated to treatment, whereas the independent Safety Monitoring Committee considered several possibly or probably treatment-related. Nevertheless, the imbalance in serious and fatal events is consistent across adverse-event categories and constitutes a randomized safety signal for this specific strategy, which should not be automatically generalized to other strategies, doses, or populations (**Table 3**).

**Table 3:** What TREASURE establishes, suggests, and does not establish.

Question	TREASURE evidence	Interpretation
Should conventional-dose TRT during maintenance be used routinely in patients selected by conventional criteria (ECOG 0–1, no progression after induction)?	Excess severe/fatal toxicity (SAE 61.3% vs 18.2%; fatal AEs 19.4% vs 3.0%); early halt	Not supported
Does TRT improve overall survival?	HR, 1.55 (95% CI, 0.90–2.69); trial underpowered after early halt	Not demonstrated
Does TRT reduce progression?	Fine-Gray analysis (death without progression as competing event): 1-year CIF of radiologic progression at any site 80.6% vs 90.6%; subdistribution HR, 0.692 (95% CI, 0.415–1.154); P = .251; death without progression 12.9% vs 3.1%	Numerically lower, not significant; overall progression only; no evidence of reduced intrathoracic progression
Is toxicity related to TRT?	Randomized safety signal, consistent across SAE and treatment-related AE categories	Consistent randomized signal; attribution of individual deaths uncertain
Is lymphocyte depletion involved in the toxicity?	Marked, lymphocyte-specific, temporally linked to TRT delivery	Biologically plausible candidate mechanism, not proven causal
Is baseline DLCO predictive of fatal risk?	Relatively reduced in the study population; lower among the 6 fatal cases in the TRT arm (n too small for validation)	Exploratory only; distinct from performance status
Is dose to blood-rich tissue (EDRIC) predictive?	Numerical proximity of total-body dose to reported thresholds in a separate cohort	Hypothesis-generating, unvalidated
Does timing determine toxicity?	Cross-trial contrasts confounded; TREASURE’s own within-trial analysis found no association	Unresolved for post-induction TRT; entangled with dose and patient selection

Radiotherapy was followed by a marked reduction in circulating lymphocytes: the proportion of patients with counts below the normal range diverged abruptly at the cycle coinciding with radiotherapy and remained higher through the fourth maintenance cycle, whereas total leukocyte and neutrophil counts were not affected to the same extent (12) (**Table 4**). This is biologically plausible, because circulating lymphocytes repeatedly pass through irradiated blood vessels and thoracic lymphoid structures, and a similar excess of decreased lymphocyte counts among irradiated patients was reported in the non-randomized adebrelimab study (33). Whether lymphocyte depletion contributed to the infectious and respiratory complications observed in TREASURE remains unknown; it is a testable mechanism rather than an established explanation (24).

**Table 4:** Absolute lymphocyte count during the first four cycles of atezolizumab maintenance in the TREASURE safety population, by treatment arm.

Timepoint	Below normal range, arm A (TRT + atezolizumab)	Below normal range, arm B (atezolizumab only)	P value	Comment
Baseline	25.8%	15.2%	.31	Arms balanced before TRT start
Cycle 1	25.9%	20.0%	.61	Difference not yet apparent
Cycle 2	91.7%	13.6%	<.001	Abrupt divergence coinciding with TRT delivery
Cycle 3	77.8%	26.7%	.004	Persistent depletion in arm A
Cycle 4	69.2%	8.3%	.002	Depletion still present at last assessed cycle

Adapted from Bozorgmehr et al., JAMA Oncology, 2026 (12).

Baseline DLCO was relatively reduced in the TREASURE population, and among patients in the radiotherapy arm who died of adverse events, baseline DLCO was lower, estimated total-body radiation exposure was modestly higher, and gross target volume was numerically larger (12). The investigators identified no risk factor for fatal toxicity beyond baseline DLCO, and these observations rest on six fatal cases. The estimated dose delivered to circulating immune cells (EDRIC) has been associated with lymphocyte depletion and outcome in other ES-SCLC cohorts (25), but TREASURE does not allow a threshold to be defined; DLCO, gross target volume, and EDRIC are therefore hypothesis-generating candidates rather than predictors.

TREASURE was stopped early and was not powered for efficacy: overall survival was numerically shorter with radiotherapy (HR, 1.55; 95% CI, 0.90–2.69), and conventional progression-free survival was similar between arms (12). In response to a methodological letter, the investigators performed a Fine-Gray competing-risks analysis in which radiologically documented progression was the event of interest and death without progression the competing event (16, 17). The 1-year cumulative incidence of radiologic progression was numerically lower with radiotherapy (80.6% vs 90.6%; subdistribution HR, 0.692; 95% CI, 0.415–1.154), but the difference was not statistically significant (P = .251), whereas death without documented progression was numerically more frequent with radiotherapy (12.9% vs 3.1%). This analysis concerns progression at any site: although time to intrathoracic progression was prespecified as a secondary endpoint in the protocol (12), the analysis reported in the correspondence addressed overall progression, and no statistically significant result for intrathoracic progression has been reported. The investigators interpreted these data as indicating that disease control with radiotherapy was at least as good as without it and that the excess mortality was mainly related to toxicity (17); this is a plausible interpretation, but not a statistically demonstrated effect. Moreover, when the competing event is more frequent in one arm, a lower cumulative incidence of progression in that arm may partly reflect patients dying before progression could be documented. The analysis therefore shows a numerically lower, non-significant cumulative incidence of overall progression and does not constitute evidence of reduced intrathoracic progression. **Table 3** summarizes what TREASURE establishes, suggests, and does not establish.

## 6 Candidate determinants of the therapeutic index

The divergent results of the prospective studies point to four interrelated questions: who should receive thoracic radiotherapy, when it should be delivered, which dose and fractionation should be used, and how much disease should be treated (**Figure 1B**). None has been tested in isolation, and cross-trial comparisons cannot disentangle them because they varied simultaneously. We therefore consider them candidate determinants of the therapeutic index rather than established predictors of benefit or toxicity.

### 6.1 Patient selection

No prospectively validated strategy for selecting patients for thoracic radiotherapy currently exists, and conventional clinical criteria do not explain the divergent safety findings of the post-induction studies. TREASURE and SAKK 15/19 both enrolled patients with ECOG performance status 0–1 and without progression after induction chemo-immunotherapy (12, 13); nevertheless, serious and fatal adverse events were markedly more frequent with radiotherapy in TREASURE, whereas no severe radiotherapy-attributable toxicity was reported in SAKK 15/19. Performance status is therefore unlikely to account for this contrast. Performance status should, however, be distinguished from other indicators of frailty and functional reserve that it does not capture. In TREASURE, baseline DLCO was relatively reduced across the study population, and lower DLCO was associated with fatal adverse events in the radiotherapy arm in an exploratory analysis (12); pulmonary reserve has not been reported in a way that allows comparison with SAKK 15/19 or the other studies. Other differences may also contribute, including randomized versus single-arm design and the resulting ascertainment and attribution of adverse events, dose and fractionation, the checkpoint inhibitor used, and target volume, but none can be isolated by cross-trial comparison. The practical implication is that eligibility criteria based on performance status and response to induction may be insufficient to identify patients at risk of serious toxicity when thoracic irradiation is combined with checkpoint blockade, which supports the prospective evaluation of measures of functional reserve, such as DLCO, together with tumor volume and estimated immune-cell exposure.

The potential benefit of thoracic radiotherapy may also not be uniform. In CREST, benefit appeared smaller in patients without residual intrathoracic disease, and a secondary analysis suggested focusing more intensive radiotherapy on patients with fewer than three distant metastases (3, 31). Retrospective studies in the chemo-immunotherapy era have similarly suggested that patients with controlled extrathoracic disease and persistent thoracic disease may represent a relevant population for investigation, although these analysis remain vulnerable to selection and immortal-time biases (7, 26).

Pulmonary reserve is therefore a leading candidate. The DLCO association in TREASURE rests on six fatal events and cannot be used as a selection criterion, but it supports the hypothesis that patients with limited pulmonary reserve may tolerate thoracic irradiation during checkpoint blockade less well, even when their performance status is preserved. Other candidate variables include depth of response, extent of residual thoracic disease, number and control of extrathoracic metastases, and the feasibility of adequate target coverage without excessive normal-tissue exposure (13, 26, 27, 31) (**Table 1**). These should be regarded as enrichment and stratification variables for future trials rather than as criteria for clinical decision-making. Future studies should also distinguish prognostic factors, which identify patients with a generally more favorable course, from predictive factors, which identify patients more likely to derive a net benefit from thoracic radiotherapy; the latter remain an unmet need.

### 6.2 Timing

Current evidence does not identify a preferable treatment window. Timing operates at three levels: the phase of treatment (induction or maintenance), the relationship to individual checkpoint-inhibitor doses, and the interval after induction. TRIPLEX shows that early concurrent conventional-dose radiotherapy during induction does not improve survival and may increase toxicity (11), whereas the contrast between TREASURE and SAKK 15/19, which used similar post-induction sequences, argues against attributing the TREASURE signal to maintenance timing alone (12, 13); within TREASURE, concurrent versus sequential atezolizumab administration was not associated with adverse events, although numbers were small (12). Outside ES-SCLC, concurrent durvalumab with chemoradiotherapy in PACIFIC-2 was associated with more fatal adverse events (13.7% vs 10.2%) and CheckMate-73L tested a related concurrent strategy, whereas durvalumab given after chemoradiotherapy improved survival in limited-stage SCLC (19–21); these data provide context but cannot be directly extrapolated to ES-SCLC.

### 6.3 Dose and fractionation

Dose and fractionation are plausible determinants, but no optimal regimen has been identified (**Table 2**). SAKK 15/19 used a higher total dose than TREASURE without a comparable safety signal, suggesting that total dose alone does not explain the divergent outcomes (12, 13), and in the adebrelimab study survival did not differ by biologically effective dose above or below 60 Gy, although this comparison was small and non-randomized (33). Fraction size may influence both normal-tissue toxicity and lymphocyte exposure, but no prospective study has compared fractionation schedules while keeping timing, treatment volume, and patient characteristics constant.

### 6.4 Treatment volume

Treatment volume may influence the therapeutic index independently of the prescribed dose: greater radiation exposure of circulating immune cells has been linked with lymphocyte depletion and outcome, and TREASURE suggests that larger target volumes and greater estimated total-body exposure may be associated with fatal adverse events (12, 25). Limiting volume cannot, however, simply mean reducing target coverage, because more complete coverage of residual thoracic disease has been associated with better outcomes than incomplete consolidation (26). Treatment planning must therefore balance meaningful coverage of residual disease against exposure of the heart, lungs, blood-pool compartments, and thoracic lymphoid structures. A lower prescribed dose could still entail substantial immune-cell exposure if a large volume is treated, whereas a higher dose might be feasible in a limited target; these possibilities remain untested hypotheses.

## 7 The consolidation paradox

The improved systemic control achieved with chemo-immunotherapy creates a potential tension for thoracic consolidation. If residual intrathoracic disease remains an important source of progression (6), the value of local control could increase; however, the only randomized evidence that thoracic radiotherapy reduces intrathoracic progression comes from CREST, in the pre-immunotherapy era (3). At the same time, the integration of thoracic radiotherapy with immune checkpoint blockade may narrow the safety margin, as suggested by TRIPLEX and TREASURE in their respective settings (11, 12). We refer to this hypothesized tension as the Consolidation Paradox. The term does not imply that cTRT should be added to modern systemic therapy, and it does not rest on evidence that thoracic radiotherapy improves thoracic control during chemo-immunotherapy, which has not been demonstrated. Rather, it describes a clinical situation in which the potential value of local consolidation and the difficulty of delivering it safely may increase together.

The relevant question is therefore not simply whether cTRT should be used, but under which treatment conditions it might provide meaningful thoracic control without compromising the benefits of systemic therapy. Current evidence raises this question but does not yet provide an answer.

## 8 Implications for future trials

Post-induction randomization is already being tested. NRG Oncology LU007 (RAPTOR; NCT04402788) is a randomized phase 2/3 trial in which patients with stable disease or partial response after platinum-etoposide plus atezolizumab are assigned to atezolizumab maintenance with or without consolidative radiotherapy to up to five thoracic and/or extrathoracic sites (20, 30, or 45 Gy) (34). Randomization is stratified by the number of irradiated sites, response to induction, and performance status; the primary endpoints are progression-free survival (phase 2) and overall survival (phase 3), with exploratory analysis by number of lesions and by completeness of consolidation. RAPTOR opened to accrual in August 2020 with a target accrual of 324 patients and, according to NRG Oncology, has been temporarily closed to accrual since September 1, 2026; results are not yet available (34, 35). It remains a key randomized trial for evaluating post-induction consolidation within the framework proposed here. Its design differs from TREASURE in including extrathoracic targets and allowing ECOG performance status 2. The recommendations below are therefore intended to complement RAPTOR and future trials rather than to propose a new design.

Stratification should prospectively consider performance status, residual thoracic disease, number and control of extrathoracic metastases, and pulmonary reserve, including baseline DLCO, recorded separately from performance status; depth of response and molecular subtype may be explored as enrichment variables. Radiotherapy should be standardized as far as possible, with predefined target volumes and dose constraints and systematic recording of lung, heart, esophageal, and blood-pool exposure. Because TREASURE raises the possibility that radiation exposure of circulating immune cells contributes to lymphopenia and toxicity, EDRIC or related measures could be incorporated prospectively as exploratory variables, and lymphocyte-sparing strategies should be tested as hypotheses rather than assumed as treatment constraints (24, 25).

Timing should be defined and recorded by treatment phase and relative to individual checkpoint-inhibitor doses. Competing-risks analysis should prespecify the cumulative incidence of radiologic progression and of death without progression alongside overall survival and conventional PFS, with thoracic progression reported separately, because PFS combines processes that may move in opposite directions (17, 28, 29); thoracic control, patterns of failure, respiratory function, and patient-reported outcomes should also be collected.

TREASURE also offers lessons for safety monitoring: its interim safety rule focused on grade 3 or greater pneumonitis, yet only one such event occurred among the first 23 patients while fatal adverse events accumulated through other mechanisms. Trials combining thoracic radiotherapy with checkpoint blockade should therefore use all-cause serious-adverse-event stopping rules and conservative effect-size assumptions and, where appropriate, include a safety run-in before larger randomized comparisons.

## 9 Conclusions

Current evidence does not support routine cTRT in patients with ES-SCLC receiving chemo-immunotherapy. The randomized phase 3 TRIPLEX trial shows that early concurrent thoracic radiotherapy during induction does not improve survival and may increase toxicity, and TREASURE provides a randomized safety signal for conventional-dose radiotherapy during atezolizumab maintenance in patients selected by conventional clinical criteria. Neither trial determines whether post-induction consolidation could have a favorable therapeutic index with different patient selection, including measures of functional reserve, or with a different technique, and no randomized evidence shows that thoracic radiotherapy improves thoracic control or survival when added to chemo-immunotherapy. This review is narrative, relies on cross-trial comparisons, and includes data from a conference presentation and from ongoing trials; its framework is intended for hypothesis generation and trial design rather than as a clinical recommendation, and RAPTOR/NRG-LU007 will provide the next key answer on post-induction consolidation.
